# Supplementary material for: FluoRNT: A robust, efficient assay for the detection of neutralising antibodies against yellow fever virus 17D
Source: PLoS One. 2022 Feb 9;17(2):e0262149. doi: 10.1371/journal.pone.0262149 (PMC8827462; doi:10.1371/journal.pone.0262149)
Supplement: S1 Note — (DOCX) [file pone.0262149.s001.docx]

**S1 Note: Notes on the definition, use and inter-comparison of assay titres generated with different assay types**.

It should be noted that antibody titre values are not absolute quantities, but rather are values on a scale dictated by the infection equivalent in use by the assay, with the requirement that they should systematically reflect neutralising antibody concentration. The actual titre determined by one or another assay therefore is not strictly comparable as they measure different effects (first round of reporter expression only, first round conversion to foci, foci conversion to plaques), which are differentially subject to different biologically variable steps (from the virus stocks prepared, to infectious events and cycles in living cells). FluoRNT and FRNT titres were found to be similar but not identical. Therefore, matching the endpoint of a commonly used but technically inferior gold standard assay should not be a success criterion for assays that are developed as improvements of the latter. Instead, data quality and reproducibility as well as practicality and objectivity of analysis, and therefore also confidence in titres, should be determined, as was shown in this work.

There is no harmonised system for reporting titres, despite WHO guidelines to use titre dilutions corresponding to EC_80_ of a sigmoidal dose-response fit (80% protection), which we use in this study and abbreviate as the "ED_80_" dilutions. Note however that, assuming consistent values for sigmoidal "top" (100%), "bottom" (0%), and similar values for Hill coefficients, EC*_x_* values will be consistently related to EC_80_ by translation through a constant and comparatively small value of log(dilution), for any value of *x* (e.g. 50 or 75), such that trends in patient EC values within a cohort should be preserved (i.e. interpretation of assay result should not depend on *x* for any reasonable value of *x* taken for analysis).
