# Supplementary material for: FluoRNT: A robust, efficient assay for the detection of neutralising antibodies against yellow fever virus 17D
Source: PLoS One. 2022 Feb 9;17(2):e0262149. doi: 10.1371/journal.pone.0262149 (PMC8827462; doi:10.1371/journal.pone.0262149)
Supplement: S1 Table — Costs are based on one full 96 well plate full of samples (e.g. serum of 0, 7, 14 and 28 dpv) assayed with FRNT against YF-17D or YF-Venus, or FluoRNT. (PDF) [file pone.0262149.s002.pdf]

	**FRNT 17D**	**FRNT Venus**	**FluoRNT**
MCS overlay	2.11 €	2.11 €	
preparation of cells	4.47 €	4.47 €	4.06 €
staining	48.81 €<br>(anti-flaviviral envelope protein antibody, HRP-conjugated secondary antibody, DAB substrate)	25.41 €<br>(anti-GFP antibody, HRP-conjugated secondary antibody, DAB substrate)	1.00 €<br>(fixable viability dye)
**sum per 96-well plate**	**55.39 €**	**31.99 €**	**5.06 €**
