# Supplementary material for: FluoRNT: A robust, efficient assay for the detection of neutralising antibodies against yellow fever virus 17D
Source: PLoS One. 2022 Feb 9;17(2):e0262149. doi: 10.1371/journal.pone.0262149 (PMC8827462; doi:10.1371/journal.pone.0262149)
Supplement: Attachment — Submitted filename: Scheck (Thorn-Seshold)_FluoRNT_Response to Reviewers.docx [file pone.0262149.s010.docx]

| **PONE-D-21-23143**  ***FluoRNT: A robust, efficient assay for the detection of neutralising antibodies against yellow fever virus 17D***  Dr. Roques | Dr. Julia Thorn-Seshold  Faculty of Chemistry and Pharmacy  Ludwig Maximilian University Munich, Germany  [julia.thorn-seshold@cup.lmu.de](mailto:julia.thorn-seshold@cup.lmu.de) | |  |
| --- | --- | --- | --- |
|  | | 29 Nov 2021 | |

Dear Dr. Roques, Dear Reviewers,

Thank you for the thorough reviews of our manuscript, and for the opportunity to submit this revised version to PLOS ONE, which we feel covers all points raised during review.

The FluoRNT assay we present is a reliable, robust and precise neutralisation assay that can be easily implemented in a lab environment; furthermore, the assay principle and workflow can be adopted to other viruses and allow the detection of neutralising antibodies independent of cytopathic effects or need for immunostaining. We feel that our substantial developments in assay speed, robustness, precision, practicality and cost render FluoRNT of broad interest to the research communities of virology, immunology and systems biology making it an excellent fit to the scope and aims of the journal.

Our point-by-point responses to the reviews are detailed below. In brief, we have clarified language and definitions of assay properties, as well as which comparisons were done experimentally vs. which were done with reference to standard practice in the literature; streamlined the introduction/results/discussion to be less redundant; added more discussion of other methods; and expanded our discussion of the use of the FluoRNT assay to give outlook for other flaviviruses.

All issues raised by the editor to meet journal requirements are also addressed: We have formatted the manuscript and named all files according to PLOS ONE’s style requirements (deleted the author summary, the financial disclosure and author contribution sections as well as the figures from the main manuscript file and uploaded all figures as separate files). We have included the source for Vero and BHK-21 cell lines in the manuscript (line 168).

Furthermore, we have double checked the financial disclosure and the funding information sections to make sure all grant numbers match. Some of the grants given in the financial disclosure statement in the manuscript do not have grant numbers, but we would still like them to appear in the manuscript if possible. Figures have not been changed.

Please do not hesitate to contact me for any questions or requests you may have.

We look forward to your feedback and comments on our revised manuscript, which is not under other consideration for publication elsewhere and has not been published elsewhere.

With best wishes from Munich,

Julia Thorn-Seshold, on behalf of all the authors

--------------------

**Reply to Reviews**

**Reviewer #1:** In the manuscript Scheck MK et al., the authors describe a neutralization assay (NT assay) for yellow fever virus (YFV) using a recombinant YFV expressing the fluorescent reporter protein Venus. The authors describe the performance of this NT assay in detail and compare the individual test steps to the protocols for plaque reduction neutralization (PRNT) and focus reduction neutralization assays (FRNT). To validate their fluorescent YFV virus-based assay (named FluoRNT), they used over 30 sera from human YFV vaccines and determined the neutralization titers resulting in 80% reduction in comparison to a focus reduction neutralization assay (FRNT). Detailed statistical analyses and dose response fitting analyses were performed to determine the accuracy and comparability of this assay. According to their data, their assay is comparable or even better with the advantage of being faster in comparison to FRNT assays. Experimental comparison to a plaque reduction neutralization assay (PRNT) was not performed, the procedures where only compared descriptive. To improve the manuscript, the authors should address the following points:

Major points:

1. Line 112 ff: for description of PRNT and FRNT – do the authors only refer to YFV assays or these assays in general? Probably only YFV – but still please specify.

We refer primarily to YFV and made that clearer in this section of the manuscript. But of course, the same principles and considerations apply similarly to other viruses as stated in lines 113 and 129 ff. PRNT and FRNT described here for YFV work the same way for NTs of any other viruses that form plaques (enabling PRNT) or for which antibodies exist to detect viral infection (enabling FRNT).

2. The protocols the authors describe for PRNT and FRNT seem to be lab specific protocols (and might also depend on the cell line used) since in other publications for YFV PRNT or plaque assays are done in 3 days compared to 4-5 days as mentioned in the manuscript (line 118). Similar is probably also true for the FRNT, which is described to be done in 96 well plates which seems a rather small format, whereas the PRNT is done in 6-12 well format. Please comment and clarify better in the manuscript (since references for the described assays are missing).

Yes, the assay protocols are described as we use them in our lab. Published YFV FRNT/PRNT protocols vary slightly from lab to lab (e.g. both shorter and longer timelines depending on the cell line and the virus stock used); however, the time lines are representative of typical procedures and we have added a more general commentary on this (lines 98ff). Thank you for pointing out the missing references; we have added references for these timelines (lines 120, 131 and 135).

3. L105: ‘The most commonly used NT assays are both reduction neutralization tests’. This sentence does not make much sense since every NT assay is a reduction neutralization assay.

I also would consider PRNT and FRNT rather similar assays (both with infectious virus) just with different read out modalities. Please adjust accordingly.

Yes, thank you for catching it, we have fixed it (line 92 f.); and yes, PRNT and FRNT are indeed similar from the setup, as we say (line 129). We describe them sequentially since (a) they are the most widely used neutralisation tests, so in this way we address the target readership no matter which assay they use; and (b) showing the advances in our paper relies on understanding the influence of *readout modality* on the precision, accuracy, and practicality of assays: so, a more detailed description of the two assay readouts to us seems adequate.

4. line 194: the authors mention that the yellow fluorescence can be seen after 3-4 days. This seems rather late. What is the titer of the YFV-Venus virus used for the studies? How does it grow in comparison to the wild-type virus?

Thank you for the opportunity to clarify this. After infection with YF-Venus, yellow fluorescence is seen within a day, as we state in e.g. line 576 ff. and illustrated in Supporting Video 1. This should match the reader’s and reviewers’ expectations. Line 194 (now line 191) mentioning “3-4 days” instead referred to electroporating cells with YF-Venus-RNA for YF-Venus virus production, which cannot be compared to how the vaccine strain grows. We edited line 191 to make this clear to the reader.

Please note that each assay type uses different measures of infectivity (e.g. FRNT => FFU, PRNT => PFU), as they operate on different time scales and have different readouts into which the functional infective units have to translate. The relevant descriptor for the virus stock in FluoRNT is the percentage of Venus+ cells obtained 24 hours post inoculation. In our protocol we aim for approximately 25% Venus+ cells (line 269); to achieve that we typically had to use approximately 5000 PFU or 2875 FFU/well/per 25.000 Vero cells of our YF-17D-Venus Stocks. That makes the amount of virus used in the FluoRNT 22-fold more concentrated than in the FRNT (line 270 ff.). As described in the section on virus stock production (lines 195 ff.) the YF-17D-Venus stock used for this study propagates somewhat slower than the YF-17D stock derived from the Stamaril vaccine that was used for this study.

5. line 334: the authors classify their FluoRNT assays as ‘single-round of infection assay’. This wording is confusing and should not be used since (1) virus release for YFV rather already starts at 8-12 hours post infection resulting in secondary infections until 24 h post infection. Thus ‘single-round of infection assay’ would not be correct. And (2) the term rather implies the use of real single-round infectious particles that really are only able to make one round of infection which were not used in this case.

Thank you for the opportunity to clarify what for our paper is a very important point. We have modified the manuscript to define “single round of infection” more precisely (lines 147, 336, 533 ff. and 576 ff.). FluoRNT readout is reachable when the first round of cells has become infected and express sufficient Venus reporter for measurement (see Fig S1 and Video S1). Regarding part (1): Video S1 particularly highlights the time-separation between the waves of reporter expression in the first and second rounds of infection. Given our timeline of measurement at 24 h and the somewhat slower kinetics of the YF-17D-Venus stock compared to the YF-17D stock, we are confident that we are indeed only detecting first-round infected cells. Regarding part (2): yes, technically YF-17D-Venus can progress to multiple rounds of infection, but since conceptually the FluoRNT assay neither requires subsequent rounds of infection, nor runs long enough for them to happen, we agree that “first” is a better description than “single”, implying there could be second, third, fourth rounds of infection, but they are not needed for the assay readout.

6. line 337 ff: Description of antibody production after vaccination rather belongs to introduction than to results part.

Thank you for the suggestion. However, we do feel it is important to mention this in the results part. Understanding the antibody production time course is essential to interpret results from the different post vaccination days. We would therefore rather leave it in the results section than making it a part of the general introduction to NTs and motivation for the FluoRNT assay (now line 339 ff.).

7. Line 359ff: This whole section is only a descriptive comparison of the assays and not a real comparison with experiments. This said – the abstract also implies that comparative analyses have been performed for both FRNT and PRNTs. However, PRNTs were not performed in comparison to FRNTs. Either the authors add them or they should make it clearer that only comparison to FRNTs was performed. The whole paragraph should be deleted in the results section and moved to the discussion, where lots of the descriptions (advantage of assay) are repeated anyway.

Thank you for the constructive suggestions. (1) We have streamlined this section (now line 366 ff.) and incorporated most of it in the discussion part. (2) We have made it clear now, that our direct experimental comparisons were only done between FRNT and FluoRNT, and comparisons with PRNT are purely descriptive (lines 370 ff.). That being said, as the reviewer also stated (item 3), FRNT and PRNT are in many ways such similar assays that we feel this experimental comparison is sufficient to highlight the benefits of FluoRNT compared to both assays: and all the more so, since FRNT is in nearly all practical respects the “better” assay than PRNT (faster, better data quality, less by-eye counting, etc.), so we feel our comparisons to FRNT are the more stringent ones that could be chosen.

8. The authors only mention PRNTs and FRNTs as alternative neutralization assays. However, other NT assays have been described as well for flaviviruses like flavivirus pseudotype viruses with GFP as read out or flavivirus single round infectious particles with luciferase as read out. The authors should mention them and compare them to their assay as well in the discussion.

Correct, we agree and implemented comparisons with other neutralisation assays as suggested (e.g. cited other advances on the PRNT- and FRNT-type assays, including the Luciferase reporter assay published by Matsuda et al. and the pseudotype reporter assay by Mercier-Delarue et al.; lines 631 ff.). We feel that the current state of the manuscript is now fair, by referencing these additional methods appropriately, yet keeping the reader’s focus and reserving the most discussion space for the most widely used neutralisation tests (with which we have previously worked).

9. What about cross reactivity? Have the authors also tried sera against other viruses in comparison of both assays?

A very good point, thank you. Our patient cohort is naïve for all flaviviruses except for TBEV. TBEV is endemic in southern Germany and about half of the vaccinees had been previously vaccinated against TBE. Anti-YFV titres of previously TBEV vaccinated participants do not differ depending on whether they were measured by FRNT or FluoRNT, suggesting, that FluoRNT is as robust to cross-reactivity as FRNT is. Data how previous vaccination influences the immune response to the YF-17D vaccine forms part of a larger study on viral cross-reactivity that will be published elsewhere and is therefore not given in this manuscript. However, in lines 647 ff. we have now included a statement to cross-reactivity and describe this equivalent robustness towards TBEV of FRNT and FluoRNT.

10. Line 591: the authors mention that the FluoRNT is robust against variations in the inoculum when using sucrose-gradient purified stocks. Does it mean that there are variations if using unpurified virus?

An important question. From day to day lab work we know that there are stock-dependent variations for both types of stocks purified as well as unpurified However, conceptually, it is of course easier to claim and show robustness between purified stocks where most contaminants are eliminated, and possibilities for variations are restricted. So, in general, we expect that any assay can benefit from using purified virus if possible. We have made it clearer in the manuscript now, that this is not a FluoRNT-specific recommendation (line 616 ff.). When stock-dependent data for FluoRNT are plotted on the same scale as the same stock-dependent data acquired using FRNT, our conclusions regarding the assay’s precision and data quality stay true regardless whether unpurified or purified virus stocks were used (as shown in Supporting Figures S2 and S4). Therefore, we feel confident to claim that the FluoRNT is essentially robust to the use of unpurified virus, at least in our hands.

Minor points:

1. line 89: which ‘four continents’? Reference?

Yes, this should have been just Africa & South America, changed and referenced (now line 75).

2. NT80 is more commonly used than ED80 for NT assays

We have clarified additionally in line 91, that we write ED for what is also called EC or NT.

3. List of costs might be relative as other antibodies/self-made antibodies might be used.

True; though we find it more helpful to give a typical estimate rather than not to give one, and we consider our estimation quite reasonable; given that cost is just one of the factors we discuss that need to be weighed when choosing an assay, we would like to keep it as it is.

4. Is it really an anti-GFP antibody that is used for detection of Venus?

Yes, since Venus is a variant of GFP, the 4-9 mutations do not affect the antibody binding site.

5. Line 594: adaptation to other viruses is mentioned. The authors should comment on the biosafety levels, since for some other reporter flaviviruses BSL3 laboratories might be necessary.

Yes, thank you, we noted this in the manuscript line 629.

In conclusion, we thank Reviewer 1 for her/his detailed reading and suggestions which we have addressed with the described manuscript changes.

--------------------

**Reviewer #2:** The authors present an FNRT method for rapid screening of neutralizing antibodies to the 17D virus. The results are clearly presented and convincing as an alternative method. FNRT has already been used and proposed by other teams for other viruses instead of PFU. Despite this last point the manuscript meets the publication criteria of PLosOne to my point (PLOS ONE does not evaluate manuscripts based on perceived significance or readership) and can therefore be accepted as is.

We thank the reviewer for this positive evaluation.
